# Evaluation of Fusarium Head Blight Resistance Through a Genome-Wide Association Study in CIMMYT and South Asian Wheat Germplasm

**DOI:** 10.3390/pathogens14050490

**Published:** 2025-05-16

**Authors:** Rupsanatan Mandal, Xinyao He, Gyanendra Pratap Singh, Muhammad Rezaul Kabir, Arun Kumar Joshi, Pawan Kumar Singh

**Affiliations:** 1 Department of Genetics and Plant Breeding, Nagaland University, Medziphema 797112, India; rup.biotech@gmail.com; 2 International Maize and Wheat Improvement Centre, Texcoco 56237, Mexico; x.he@cgiar.org; 3 ICAR-Indian Institute of Wheat and Barley Research, Karnal 132001, India; gp.singh@icar.gov.in; 4 Bangladesh Wheat and Maize Research Institute, Dinajpur 5200, Bangladesh; rezaulwrc@gmail.com; 5 International Maize and Wheat Improvement Center (CIMMYT)-India Office, New Delhi 110012, India; a.k.joshi@cgiar.org; 6 Borlaug Institute for South Asia, New Delhi 110012, India

**Keywords:** fusarium head blight, GWAS, wheat, SNP markers, marker trait association

## Abstract

Fusarium head blight (FHB) is an important disease throughout the world due to its strong association with yield reduction, quality deterioration, and mycotoxin contamination in wheat. The use of FHB-resistant genotypes in wheat production can significantly reduce damage. The current study screened a panel of bread wheat from CIMMYT and South Asian countries for FHB resistance to identify promising genotypes useful for wheat breeding and to map the associated genomic regions and linked molecular markers through a genome-wide association study (GWAS). Spray-inoculated field experiments were conducted at CIMMYT, Mexico, over three years, and a wide range of phenotypic variations was observed. Four lines, CIM-39, CIM-29, CIM-9, and CIM-3, exhibited consistent resistance across experiments, with FHB indices ranging from 6.5 to 8.1. Genotyping was conducted using the Illumina Infinium 15 K Bead Chip, and 11,184 high-quality SNP markers were obtained and used for GWAS. Nineteen significant marker-trait associations (MTAs) were detected, among which MTAs at *Ra_c58315_265* on 1A and *Tdurum_contig102328_129* and *Ku_c20136_198* on 7B showed reproducible results, with phenotypic effects on FHB resistance of 6.05%, 3.54%, and 3.92%, respectively. Several genes associated with disease resistance were found near the significant SNPs. The identified resistant genotypes and markers may be useful in future marker-assisted breeding in wheat.

## 1. Introduction

Wheat (*Triticum aestivum* L.) is a staple food crop for 35% of the global population, with an annual production of 783.43 million tonnes in 2023–2024 [1]. By 2050, with a projected global population of 9 billion, there is an urgent need to increase wheat production to meet the growing wheat demand [2]. Severe disease epidemics under climate change scenarios pose a high risk to wheat production [3,4]. Fusarium head blight (FHB), caused by *Fusarium* spp., is a fungal disease that hinders wheat production in warm and humid regions worldwide. In the 1990s, it led to significant outbreaks in the U.S. Midwest, and Canada, resulting in substantial financial losses due to reduced wheat yields amounting to billions of dollars [5,6]. FHB is the second most devastating disease affecting wheat production in the U.S. Midwest, and Canada [7]. Notable outbreaks have been reported in most wheat-producing countries, including the top producers like China, France, India, Russia, and the United States, which collectively contribute to 50% of global production [7]. Fusarium head blight produces a range of mycotoxins, especially deoxynivalenol (DON), and ingestion of mycotoxin-contaminated grains poses a threat to global food and feed safety, as it can lead to symptoms such as nausea, vomiting, diarrhoea, weight loss, and gastrointestinal abnormalities in both humans and livestock. The U.S. Food and Drug Administration sets guidelines on the maximum amounts of DON allowed in end-use products, and grains with high DON are often associated with significant price reductions and substantial economic damages [6].

FHB management requires an integrated approach, including host resistance, fungicide application, and agronomic practices [8]. Host resistance is widely accepted as a sustainable, economically efficient, and ecologically benign strategy for managing this disease. Resistance to FHB is inherited quantitatively and can be categorised into five distinct types, as described by Mesterházy (1997) and Mesterházy et al. (1999) [9,10]. Extensive research has been conducted on two types of resistance: type I, which confers resistance to initial infection based on FHB incidence and type II, which influences disease spread within spike tissues based on FHB severity. In the last two decades, resistance sources of type III, which reduce the accumulation of DON, and type IV, which reduce the occurrence of Fusarium-damaged kernels (FDK), have gained more attention from wheat breeders, due to the growing global concern about wheat grain quality and mycotoxin contamination [10,11,12]. Recent research advances have underscored the need for integrated multi-omics strategies to better understand the diverse molecular pathways involved in wheat FHB resistance [13].

Association mapping studies have been conducted on various wheat germplasms to investigate the genetics of resistance to FHB. Kollers et al., 2013 [14] utilised 732 microsatellite markers to conduct an association mapping study on 358 European winter wheat accessions and discovered genetic loci for FHB on all wheat chromosomes, except chromosome 6B. In another study, Arruda et al. (2016) [15] identified 10 significant marker-trait associations (MTAs) on chromosomes 4A, 6A, 7A, 1D, 4D, and 7D for various FHB resistance components. The availability of high-density markers has enabled genome-wide association studies (GWAS), which have become popular for identifying QTL that contribute to different types of FHB resistance. GWAS offers higher genetic resolution due to historical recombination events and the abundant genetic variation found across a wide range of accessions. Numerous studies using bi-parental mapping and GWAS have revealed over 500 QTL on all wheat chromosomes that are responsible for various types of FHB resistance [16,17,18,19,20,21,22]. Nevertheless, only a small number of QTL have undergone fine mapping, such as *Fhb1* (*Qfhs.ndsu-3BS*) located on wheat chromosome 3BS [23], *Fhb2* (*Qfhs.nau-6B*) on 6BS [24], and *Qfhs.ifa-5A* on 5A [25], all of which were derived from the FHB-resistant Chinese wheat cultivar Sumai#3. Additional *Fhb* genes include *Fhb3* from *Leymus racemosus*, *Fhb4* (*Qfhi.nau-4B*), *Fhb5* (*Qfhi.nau-5A*), and *Fhb8* (*Qfdk.nau-7D*) discovered in the Chinese landrace Wangshuibai, *Fhb6* from *Elymus tsukushiensis*, *Fhb7* from *Thinopyrum elongatum*, and *Fhb9* from the Chinese variety Shi4185. Of these, only *Fhb1* and *Fhb7* have been successfully cloned in recent times [26,27,28]. Both genes have been utilised in wheat breeding programmes [29,30,31]. Nevertheless, it is more advantageous to incorporate resistance genes from indigenous sources through introgression, which minimises the potential introduction of unwanted genetic elements caused by linkage drag, which can have detrimental effects on crop output and end-use characteristics [30,32,33]. Furthermore, indigenous sources are better suited to local environmental circumstances than foreign genotypes.

Although South Asia is not a major FHB epidemic region, the disease incidence has been increasing in the last decades due to changes in climate and agricultural practices [34,35]. To prevent large-scale FHB outbreaks in the future, it is crucial to continue searching for and utilising sources of FHB resistance within the local gene pool, which, in combination with the introduction of major QTL through marker-assisted selection and genomic selection, will contribute to the improvement of FHB resistance in South Asia. The main goals of this study were to evaluate 174 bread wheat lines of CIMMYT and South Asian wheat germplasm (CSASWG) for FHB resistance under field conditions, as well as to identify and investigate genomic regions linked to FHB resistance.

## 2. Materials and Methodology

### 2.1. Plant Materials

**Figure 1:**
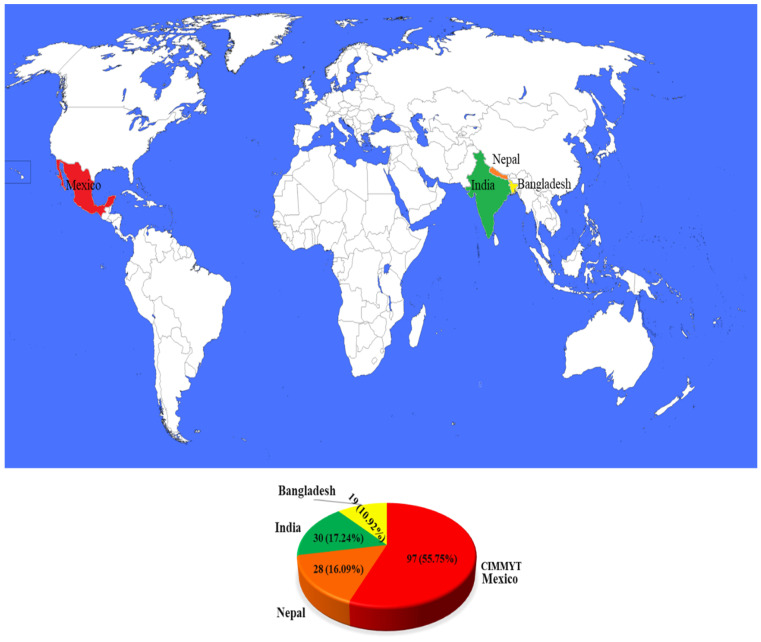
Distribution of the studied germplasm among the four countries, represented by different colours. The pie chart shows the share of the CSASWG by country.

In the present investigation, we utilised a panel of 174 CSASWG lines. Among them, 97 genotypes (CIM-1 to CIM-97) were obtained from CIMMYT-Mexico, 30 genotypes (IND-1 to IND-21, IND-28, IND-30 to IND-36, and IND-38) from India, 28 genotypes (NPL-1 to NPL-28) from Nepal, and 19 genotypes (BGD-1 to BGD-19) from Bangladesh (Figure 1). These genotypes are indicative of the current top-tier varieties and breeding lines within their respective organisations and countries (Supplementary Table S1).

### 2.2. Field Trials and Disease Scoring

**Figure 2:**
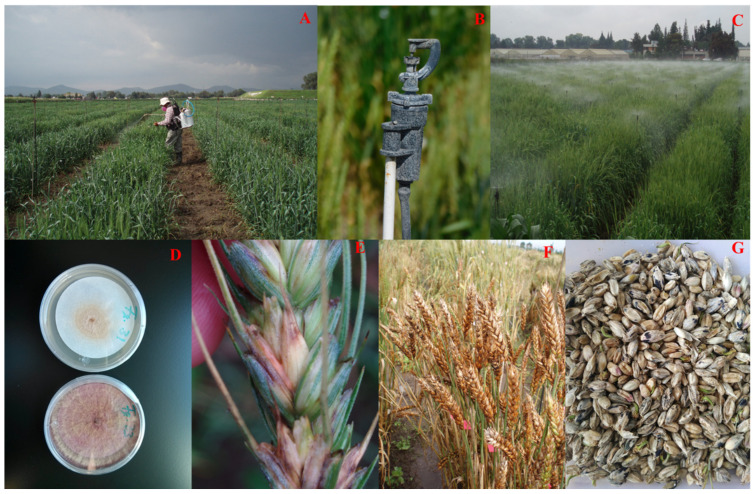
FHB disease screening at CIMMYT-Mexico. (**A**) Spray inoculation with *F. graminearum* inoculum; (**B**) Nozzle used for the automatic misting system; (**C**) Mist irrigation in the FHB screening field; (**D**) Laboratory culture of an *F. graminearum* isolate; (**E**) FHB on a wheat spike, showing symptoms of chlorosis, necrosis, and salmon-coloured mycelial mass; (**F**) A highly susceptible wheat line showing necrosis caused by FHB; (**G**) *F. graminearum* infected wheat grains.

Field experiments for FHB were conducted at CIMMYT’s El Batán experimental station in Mexico in 2019, 2021, and 2023. The station is located at an altitude of 2240 m above sea level, with coordinates 19.5° North and 98.8° West. The average annual precipitation at the station was 625 mm. The experiments were conducted during the summer season, which spans from May to September and is characterised by concentrated rainfall [36]. CSASWG accessions were planted in double rows of 1 m in length, using a randomised complete block design with two replications. Four checks were included in the experiments: SUMAI #3 (resistant), HEILO (moderately resistant), OCORONI-F-86 (moderately susceptible), and GAMENYA (susceptible). Annually, five virulent *F. graminearum* isolates were collected, analysed, and employed in a mixture for field inoculation, adhering to the procedures outlined by [37]. Spray inoculation targeted the anthesis stage of each line using an inoculum concentration of 50,000 spores/mL at a rate of 60 mL/m^2^. This process was repeated two days after the initial spray. During the period from anthesis to early dough stages, the nursery was subjected to misting from 9:00 a.m. to 8:00 p.m. with 10 min of spraying every hour. This was done to establish a humid environment that would promote the development of FHB. In the nursery, wheat-maize rotation and conservation farming methods were implemented to improve the presence of natural inoculum (Figure 2). FHB symptoms were assessed 25 days after inoculation (dpi) on 10 spikes that were marked at flowering. The number of infected spikes and symptomatic spikelets on each spike was recorded in order to calculate the FHB_index_ using the formula FHB_index_ (%) = Severity × Incidence × 100 [38]. Severity was quantified as the mean percentage of spikelets affected by the disease, whereas incidence was defined as the percentage of spikes showing symptoms. Days to heading (DH) and plant height (PH) were also scored in all experiments.

### 2.3. Statistical Analysis

The adjusted FHB_index_ values were obtained by subtracting the residuals from the linear regression model with DH and PH as covariates, using the “resid” function of R software v 4.4.1, to reduce the influence of the two traits on FHB infection, and the adjusted values were used for all subsequent analyses. Analysis of variance for FHB_index_ was performed using the “agricolae” package, and genotype-by-year interaction was estimated with “metan” package in R. Broad-sense heritability was estimated for three years using the formula H^2^ = σ^2^_g_/((σ^2^_g_ + σ^2^_g-y/_y) + σ^2^_e/_ry), where σ^2^_g_ stands for genotypic variance, σ^2^_e-y_ for genotype-by-year interactions, σ^2^_e_ for error variance, y for the number of years, and r for the number of replications [39]. The raincloud plot was projected to show the distribution of FHB_index_ values across experiments using the “ggrain” package in R software v 4.4.1. Tukey’s HSD test was performed to determine the significant differences among genotypes using PAST v 4.03 software. All bar graphs were drawn using Microsoft Excel 2021.

### 2.4. Genotyping, Population Structure, and LD Analysis

Genomic DNA was isolated from seedling leaves three weeks old using the CTAB method. DNA quality and quantity were evaluated on a Thermo Scientific™ NanoDrop™ 2000 Spectrophotometer (Waltham, MA, USA), and then sent to Trait Genetics GmbH, Germany, for genotyping using Illumina Infinium 15 K Bead Chip. A total of 11,184 SNP markers were obtained after filtering (excluding SNPs with minor allele frequencies less than 5%, missing data points greater than 30%, and those with unknown chromosome positions). The optimum number of clusters PCA was calculated using GenAlex v 6.5 tools, followed by a phylogenetic tree, kinship analysis, and linkage disequilibrium were performed using Tassle v 5.0 and R software v 4.4.1 with the rMAV package.

### 2.5. Marker-Traits Association (MTA) for FHB

Multiple Loci Mixed Model (MLMM) was used to detect SNP-FHB_index_ associations with the “Gapit” package in R, and significant markers in all models were declared with a threshold of *p* < 0.01 [40]. FHB data for the three years were used individually for GWAS, followed by pooled FHB_index_ values.

## 3. Results

### 3.1. Reaction of Genotypes Against FHB and Identification of Resistant Lines

**Figure 3:**
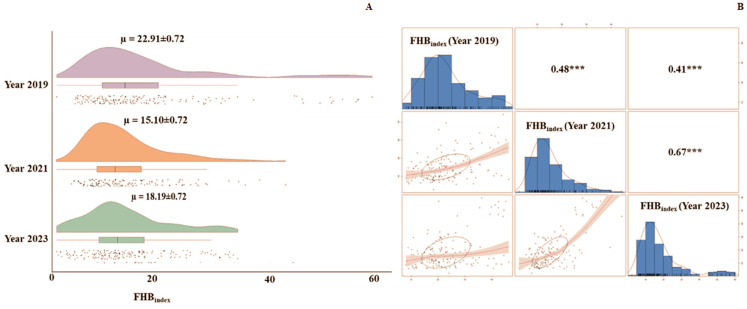
FHB infection in the CSASWG panel. (**A**) Raincloud distribution plots for FHB_index_ in the three years, with mean FHB_index_ and S.E. values indicated at the top of each plot. (**B**) Histogram distribution of FHB_index_ in individual years and correlation plots and values for the three years. “***” indicates significance at the *p* < 0.0001 level.

A raincloud plot (Figure 3A) depicts the average disease index of FHB throughout the three separate field trials in 2019, 2021, and 2023. The correlation coefficients of the FHB_index_ among the three field evaluations ranged from 0.41 to 0.67 (Figure 3B). The grand mean FHB_index_ for 2019, 2021, and 2023 were 22.91%, 15.10%, and 18.19%, respectively.

**Figure 4:**
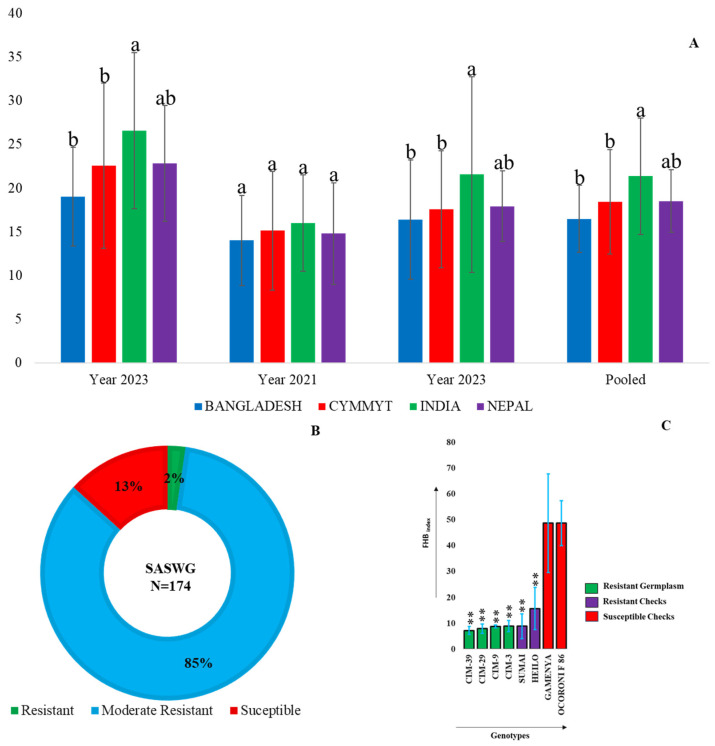
(**A**) Mean FHB indices of different country groups in individual years. The letters above the bars indicate groups that are significantly different at the 5% significance level. (**B**) Distribution of 174 genotypes according to FHB resistance. (**C**) Differences between checks and identified resistant lines. “**” indicates significance at the 1% confidence level.

The 174 wheat genotypes showed a wide range of variation, from resistant (0–9.0) to susceptible (>25). The majority of genotypes (>84.48%) had disease scores between 9.0 and 25.0, indicating a moderately resistant disease reaction. CIMMYT lines outperformed the other groups of lines, whereas the Indian varieties tested in this study exhibited generally high infection (Figure 4A). Only four genotypes had disease indices between 0 and 9.0, indicating a resistant disease reaction and 23 genotypes with FHB_index_ > 25.0 demonstrated a susceptible response (Figure 4B).

The resistant lines, such as CIM-39, CIM-29, CIM-9, and CIM-3, had a mean FHB_index_ ranging from 0.0 to 9.0 throughout all three years of field evaluation (Figure 4C). Significant effects were found for both “year” and “genotype”, whereas their interaction did not show significant effects (Table 1). The overall broad-sense heritability (H^2^) for the FHB index was 0.77.

### 3.2. Genotyping, PCA, Kinship and Linkage Disequilibrium Analysis

**Figure 5:**
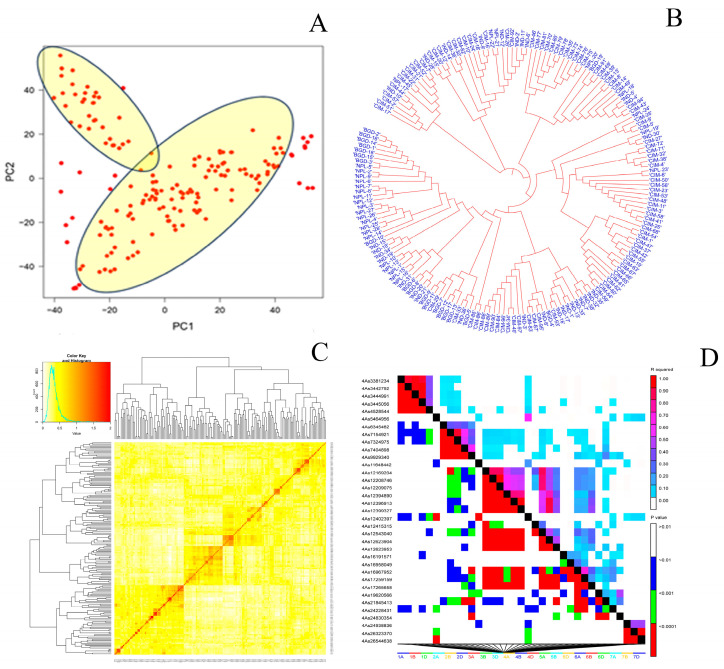
(**A**) 2D-PCA plot and (**B**) phylogenetic tree explored diversity in the 174 wheat genotypes; (**C**) Heatmap and dendrogram of Kinship matrix estimated using Van Randen algorithm based on the 11,184 SNP markers; (**D**) LD plot of wheat genome based on 11,182 SNP markers. The red colour indicates the haploblocks.

A total of 16,028 SNP markers were generated using the Illumina Infinium 15 K Bead Chip platform, producing a total of 11,184 SNP markers after excluding markers with minor allele frequencies less than 0.05, unknown chromosome positions, or more than 10% missing SNP data. Before performing GWAS, the 11,184 SNP markers were used for PCA, which separated the 174 CSASWG lines into two clusters, that is, of CIMMYT origin and other than CIMMYT (Figure 5A), which agreed well with the dendrogram analysis using the same set of SNP markers (Figure 5B). Kinship analysis also revealed significant differences between the CIMMYT genotypes and those other than CIMMYT, as seen in Figure 5C. LD plot was generated using the same set of SNP markers based on the r^2^ values for the entire genome (Figure 5D).

### 3.3. Genome-Wide Association Study

**Figure 6:**
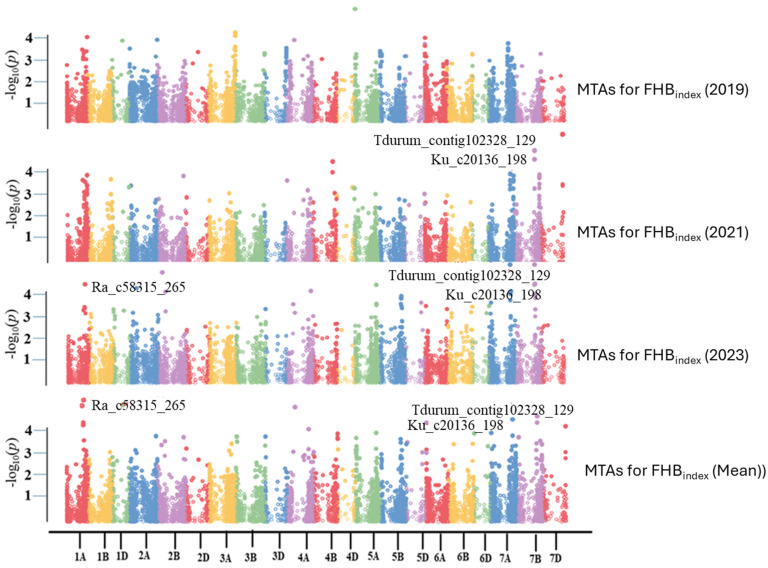
Manhattan plots based on the Multiple Loci Mixed Model (MLMM) represent −log10 (*p*-value) for SNPs distributed across all chromosomes (*x*-axis).

Four different algorithms, namely GLM, MLM, MLMM, and FarmCPU, were initially compared to select the best algorithm for association analysis. MLMM better fits the FHB disease data and was used to identify significant MTAs with a significance threshold of *p* < 1 × 10^−4^. Nineteen significant markers were identified on eight chromosomes, of which three were repeatable across different experiments and were located on chromosomes 1A and 7B (Table 2 and Figure 6). Phenotypic variations explained (PVE) by the three significant SNPs, i.e., *Ra_c58315_265* (on chromosome 1A), *Tdurum_contig102328_129* (7B), and *Ku_c20136_198* (7B), ranged from 3.34 to 6.05% (Table 2). PVEs of other significant SNPs were also low, ranging from 2.05 to 6.23% (Table 2).

**Figure 7:**
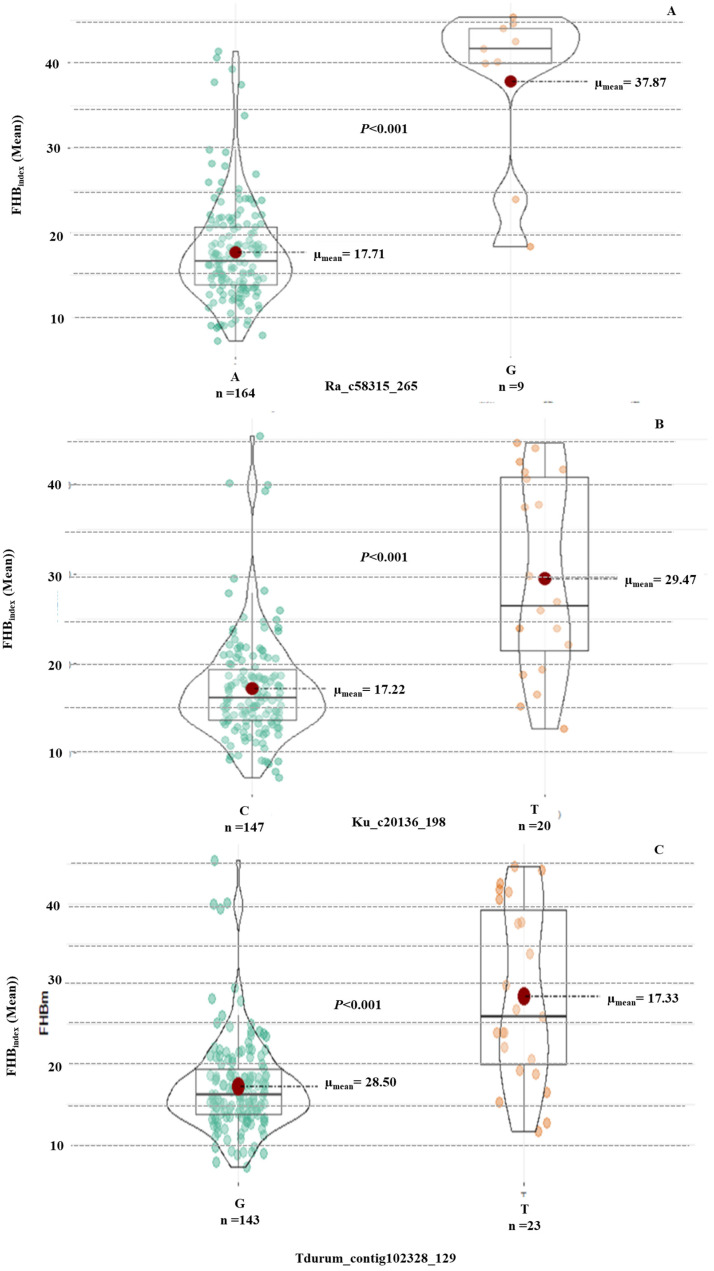
Effect of different alleles using the Wilcoxon test for (**A**) MTA Ra_c58315_265 with FHB_index_ (mean), (**B**) MTA Ku_c20136_198 with FHBindex (mean), and (**C**) MTA Tdurum_contig102328_129 with FHBindex (mean).

Haplotype analysis revealed significant differences between the resistant and susceptible alleles for the three MTAs on 1A and 7B (Figure 7). Haplotype results also indicated that the allele “A” from SNP *Ra_c58315_265*, “C” allele from *Ku_c20136_198,* and “G” allele from *Tdurum_contig102328_129* were associated with the FHB resistance mechanism in wheat. These alleles were present in all the most resistant genotypes (Table 3), suggesting an additive effect of these alleles on FHB resistance in wheat.

### 3.4. Candidate Genes for the Significant MTA

Sequences of the 19 significant SNP markers identified from GWAS were used in BLAST searches against the *Triticum aestivum* v2.2 reference genome available at Phytozome v 13 (accessed on 26.06.24) to identify candidate genes related to disease resistance mechanisms in plants. For each MTA, a two-Mb window was used to identify candidate genes, and a total of 12 genes were found (Table 4).

## 4. Discussion

FHB is a globally important wheat disease, and its incidence in South Asia has been increasing due to climate change [8,34]. Identification of genotypes with FHB resistance could be an important pre-emptive strategy for possible future epidemics in the region, and the significant markers identified through GWAS could be used in marker-assisted breeding programmes.

The observed variation in the responses of the wheat accessions over different years can be attributed to the fluctuating weather conditions, which were reflected in the significant “year” effect in ANOVA; however, no significant genotype-by-environment effect was observed for this panel. Admittedly, disease pressure in the three years was not very high, as reflected in the generally low FHB indices of the tested lines, as well as the susceptible checks that often reached 80–90% under high disease pressure. Therefore, lines with low infection in this study might be moderately resistant rather than fully resistant, as one would infer based on the FHB indices. Nevertheless, the results might still be highly relevant to the South Asian scenario with low FHB disease pressure. In a recent study on screening Indian wheat germplasm for FHB resistance, Kumar et al. (2021) evaluated 164 wheat genotypes and did not identify any resistant lines; only a few showed moderate resistance, while the rest were moderately susceptible or susceptible [35]. This is consistent with our results, although we used spray inoculation to assess a combination of type I and type II resistance, whereas they used the cotton web technique to evaluate type II resistance specifically. Heading date and plant height have long been associated with field FHB infection, which affects the precision evaluation of materials in field experiments [36]. In the current study, the two factors were used as covariates to correct the FHB index, which was used in all subsequent analyses, avoiding the confounding effects of heading and height, resulting in a better estimation of genetic resistance.

CIMMYT lines generally outperformed those from other South Asian countries in this study, and the best lines identified were all of CIMMYT origin, which was not unexpected because FHB resistance is an important target of wheat breeding at CIMMYT [37], but it has not become a major trait for South Asian wheat breeding programmes yet. Nevertheless, it is well known that the most effective FHB resistance genes/QTL like *Fhb2*, *Fhb4*, and *Fhb5,* are of very low frequencies, if not absent [41], whereas *Fhb1* has been introduced into CIMMYT germplasm, but its frequency is still low and needs to be increased [42]. Significant breeding efforts are being conducted at CIMMYT to utilise these genes along with the recently cloned *Fhb7* to improve FHB resistance, which may also contribute to South Asian wheat breeding, considering the frequent utilisation of CIMMYT germplasm as parents.

Nineteen MTAs were identified by the MLMM method in the present GWAS, which identified the highest number of MTAs on 7B chromosome, followed by 7A, 4A, and 1A. However, only three MTAs (*Ra_c58315_265* on 1A, *Tdurum_contig102328_129,* and *Ku_c20136_198* on 7B) These results revealed marker-trait relationships, indicating the presence of resistant alleles, with effects ranging from 3.54% to 6.05% for FHB resistance in the field. This resource plays a crucial role in the production of FHB resistance markers, thereby enhancing the efficacy of selecting FHB-resistant traits for variety improvement [43]. Consistent with a recent investigation, some QTLs were also reported by Shi et al., 2023 [44], on chromosome 1A (*gFHB-1A.1*, *gFHB-1A.2,* and *gFHB-1A.3*) and Chromosome 7B (*gFHB-7B*). According to meta-QTL analysis, five MQTL-related FHB indexes were reported on 1A chromosome [21].

The candidate genes linked to the identified MTAs included Glycosylase/Endonuclease III, Putative transmembrane protein cmp44E, and cysteine-rich repeat protein. Lu and Edwards (2016) and Guo et al. (2020) [45,46] reported that the cysteine-rich repeat protein has a significant role in the defence mechanism against both *Rhizoctonia cerealis* and *Bipolaris sorokiniana*, and Paudel et al. (2020) [47] reported that putative transmembrane protein is associated with *Qfhb1* in wheat. While the precise molecular consequences of the associated polymorphisms remain to be elucidated, these loci may influence resistance through regulatory or structural changes in candidate genes. Further work is needed to confirm the roles of these candidate genes in FHB resistance.

FHB resistance is a complex quantitative trait and resistance in wheat is subject to environmental conditions. The current study identified 19 MTAs, of which only three were repeatable and can be useful in future marker-assisted breeding programmes. The identified resistant genotypes may be used as parents or released as varieties in the South Asian region, considering their good FHB resistance.

## 5. Conclusions

In conclusion, this study underscores the general susceptibility of South Asian wheat genotypes to Fusarium head blight (FHB) and emphasises the importance of pre-emptive strategies like identifying resistant wheat genotypes for their utilisation in breeding. Marker-assisted breeding programmes can greatly benefit from the findings of this GWAS study, particularly the three reproducible MTAs (Ra_c58315_265, Ku_c20136_198, and Tdurum_contig102328_129) identified on chromosomes 1A and 7B. While acknowledging the variable responses of wheat accessions across years, this study highlights CIMMYT lines as good performers in FHB resistance despite the challenges posed by the low frequencies of key resistance genes. These findings provide a foundation for improving FHB resistance in South Asian wheat varieties, thereby contributing to regional agricultural resilience.

## Figures and Tables

**Table 1:** Analysis of variance for FHB_index_ of the CSASWG panel across field experiments.

Source	Df	Mean Square	F Value	*p*-Value
Year	2	5903.51	82.64	*p* < 0.001
Genotype	173	327.37	4.58	*p* < 0.001
Genotype × Year	198	76.12	1.07	0.28
Rep	1	365.05	5.11	0.02
Error	669	71.44		

**Table 2:** Markers significantly associated with FHB resistance through MLMM.

Traits	SNP	Chr	Pos	*p*-Value	Effect
FHBm	*wsnp_Ex_c11055_17927668*	5D	561706128	0.000794	3.06
FHB21	*Tdurum_contig44948_1812*	7B	699838421	0.000648	2.91
FHB23	*Tdurum_contig28176_55*	7B	544138607	0.000251	5.49
FHB23	*Tdurum_contig102328_129	7B	565742062	0.000754	3.54
FHB21	*Tdurum_contig102328_129	7B	565742062	0.000138	3.44
FHBm	*Tdurum_contig102328_129	7B	565742062	0.000491	2.83
FHB21	*Tdurum_contig10036_474*	1A	556304788	0.000521	2.61
FHB21	*RAC875_rep_c111159_57*	7A	700808659	0.000859	2.81
FHBm	*RAC875_c60218_63*	4A	217653852	0.000257	2.05
FHB23	*RAC875_c32611_347*	4A	669816688	0.000427	4.46
FHB21	*RAC875_c2456_849*	4B	562356529	0.000409	4.25
FHB23	*Ra_c58315_265	1A	473637106	0.000242	6.05
FHBm	*Ra_c58315_265	1A	473637106	0.000152	4.99
FHB21	*Kukri_c9683_723*	7A	700702592	0.000537	2.97
FHB23	*Ku_c20136_198	7B	609385718	0.000434	3.92
FHB21	*Ku_c20136_198	7B	609385718	0.000458	3.34
FHBm	*Ku_c20136_198	7B	609385718	0.000618	2.96
FHB23	*Excalibur_rep_c111660_89*	7A	585065925	4.65 × 10^−5^	6.23
FHBm	*Excalibur_c59894_97*	1A	463813596	0.000928	3.71
FHB23	*BS00059062_51*	7B	545991407	4.65 × 10^−5^	6.23
FHB23	*BS00059061_51*	7B	545991505	0.000251	5.49
FHB21	*BobWhite_c14736_188*	7B	699375055	0.000492	3.02
FHBm	*AX-94793903*	1D	291636258	0.00022	2.11
FHB23	*AX-94639168*	2B	107117765	8.98 × 10^−5^	4.11

“*” highlights the repeatable MTAs for FHB resistance.

**Table 3:** The presence of different alleles associated with FHB disease resistance observed in the most resistant genotypes.

Genotypes	Ra_c58315_265	Ku_c20136_198	Tdurum_contig102328	FHB_index_ (Mean)
	Allele (A/G)	Allele (C/T)	Allele (G/T)	
CIM-29	A	C	G	7.93
CIM-39	A	C	G	7.26
CIM-9	A	C	G	8.80
CIM-3	A	C	G	8.94

**Table 4:** Functional annotation of candidate genes associated with disease resistance.

SNP	Chr	Functional Annotation
Tdurum_contig10036_474	1A	PROTEASOME NON-ATPASE REGULATORY SUBUNIT 5-RELATED
Ra_c58315_265	1A	A/G-SPECIFIC ADENINE GLYCOSYLASE/ENDONUCLEASE III
Excalibur_c59894_97	1A	THYMIDINE KINASE 2
AX-94793903	1D	Defensin and Defensin-like DEFL family
AX-94639168	2B	Unknown
RAC875_c60218_63	4A	F-box and tubby domain-containing protein,
RAC875_c32611_347	4A	C-terminal processing peptidase/Tsp protease
RAC875_c2456_849	4B	Exocyst complex subunit Sec15-like
wsnp_Ex_c11055_17927668	5D	COP1-INTERACTING PROTEIN-LIKE PROTEIN
RAC875_rep_c111159_57	7A	histone acetyltransferase of the TAFII250 family 2
Kukri_c9683_723	7A	Transcription initiation factor TFIID subunit 1
Ku_c20136_198	7B	Putative transmembrane protein cmp44E
Excalibur_rep_c111660_89	7A	AN1-TYPE ZINC FINGER PROTEIN
Tdurum_contig44948_1812	7B	transcription initiation factor TFIID subunit 1
Tdurum_contig28176_55	7B	AN1-TYPE ZINC FINGER PROTEIN
Tdurum_contig102328_129	7B	Cysteine-Rich repeat protein
BS00059062_51	7B	Unknown
BS00059061_51	7B	Unknown
BobWhite_c14736_188	7B	Methyl-CpG binding domain (MBD)

## Data Availability

Phenotypic data are available in the Supplementary File, and the genotypic data are accessible at https://hdl.handle.net/11529/10548559 (accessed on 2 May 2025).

## Supplementary Materials

The following supporting information can be downloaded at: https://www.mdpi.com/article/10.3390/pathogens14050490/s1, Supplementary Table S1: List of genotypes with their raw and adjusted FHB_index_ values.
